# Investigating the link between frontal sinus morphology and craniofacial characteristics with sex: A 3D CBCT study on the South Indian population

**DOI:** 10.12688/f1000research.137008.1

**Published:** 2023-07-11

**Authors:** Ceena Denny, Mohana Bhoraskar, Sabiha Abdul Aziz Shaikh, Bastian T S, Nanditha Sujir, Srikant Natarajan

**Affiliations:** 1 Oral Medicine and Radiology, Manipal College of Dental Sciences, Mangalore, Manipal Academy of Higher Education, Manipal, Karnataka-575001, Mangalore, India; 2 Oral and Maxillofacial Pathology, MAHE Institute of Dental Sciences, Chalakara, Puducherry, 673010, India; 3 Oral and Maxillofacial Pathology, Manipal College of Dental Sciences, Mangalore Manipal Academy of Higher Education, Manipal, Karnataka.India-575001, Mangalore, India

**Keywords:** Frontal sinus, Craniofacial parameters, Forensic science, Cone Beam Computed Tomography, Sex determination

## Abstract

**Background:** Measurement of craniofacial parameters plays an important role in sex determination in forensic science. The present study was done using cone beam computed tomography (CBCT) scans to evaluate the morphologic structure of the frontal sinuses and compare it with the width of nasal, cranial, maxillary and mandibular width which might help us in sex determination.

**Methods:** A cross-sectional retrospective study was conducted using 142 full field of view (FOV) scans of patients archived from the department. The width of the nose, cranium, maxilla, and mandibular width was measured and compared with the frontal sinus between the two sexes.

**Results:** A paired t-test was done to compare the linear measurements for both sexes' right and left frontal sinuses. The measurements were higher in males when compared to females. There was a statistically significant asymmetry (larger dimension on the left side) of the anterioposterior (p-value of 0.012) and superior-inferior dimensions in males (p-value of 0.135). Spearman's correlation showed that frontal sinus correlated with other craniofacial parameters like nasal, cranial, maxillary and mandibular width among both sexes. The frontal sinus, nasal, cranial, maxillary and mandibular widths were higher in males when compared to females (independent t-test). Discriminant function scores showed 66-68% accuracy to discriminate sex, using the anteroposterior dimension and mandibular width.

**Conclusions:** The measurement of craniofacial parameters using CBCT can aid in determining the sex of unidentified and decomposed bodies.

## Introduction

Determining the identity of human remains through visual assessment of craniofacial parameters using linear measurements is a common practice in forensic science. Unfortunately, accidents, natural disasters, and fires can cause severe decomposition or damage to the body, making identification difficult. In these cases, anthropological knowledge is crucial in identifying the remains. The skull, being the most complex and stable part of the human body, can withstand force and assist in forensic investigations. ^1^

The frontal sinus is a crucial component of the human skull, located in the pneumatized cavity of the frontal bone. While typically paired, these sinuses are seldom symmetrical and can vary in size. Radiographically visible by the 2nd or 3rd year of life, the frontal sinus completes its growth by the age of 20. Interestingly, these sinuses are unique to each individual, even when compared in monozygotic twins. ^2^ By analyzing the shape and size of the frontal sinus, we can gain valuable insights into an individual's craniofacial parameters. This information can be used to diagnose and treat various medical conditions, including sinusitis and facial trauma. It is important to note that the morphology of the frontal sinus can vary significantly between individuals, making it a valuable tool in personalized medicine.

Radiographs are crucial in identifying anthropological structures when DNA samples and soft tissues are not accessible. Radiology plays a significant role in determining these structures, particularly when considering morphological parameters. ^3^ Cone beam computed tomography (CBCT) is a cutting-edge diagnostic imaging technique that provides a three-dimensional view of a specific region in the head and neck. This technology allows for the visualization of anatomical structures from different angles, without any superimposition, distortion, or magnification. The accuracy of measurements obtained through CBCT makes it a superior imaging modality compared to traditional two-dimensional imaging. ^4^ One of the most significant advantages of CBCT is its cost-effectiveness, as well as its ability to expose individuals to less radiation. This makes it a safer and more accessible option for patients. While CBCT has been widely used in the field of anthropology, it has recently gained more attention in the field of forensic identification. ^5^

Numerous studies have been conducted in the past using radiographs to assess the importance of the frontal sinus in determining an individual's characteristics. However, very few studies have been conducted using CBCT in the South Indian population. Our study aims to fill this gap by focusing on the South Indian population, which has distinct craniofacial features compared to other populations. Through this research, we hope to enhance the understanding of the relationship between frontal sinus morphology and craniofacial parameters in this specific population.

The purpose of this study is to evaluate the morphological structure of the frontal sinuses and compare it with the nasal, maxillary, cranial and the mandible widths. This information can be useful in identifying and determining human characteristics.

## Methods

### Ethics and consent

Ethical approval was obtained from the Manipal College of Dental Sciences' Institutional Ethics Committee (protocol ref no: 21056, dated 20/9/21). As part of the dental treatment, participants were asked to provide written consent for the use of their anonymized data in future research and publications.

### Design and sample size

A cross-sectional retrospective study was conducted after obtaining ethical clearance. 142 full field of view (FOV) scans of patients were archived from the Oral Medicine and Radiology department **.** The images selected were those of South-Indian population which included a subset of residents of the Dakshina Kannada district catering to the West coast of India.

Based on the article “Association between frontal sinus morphology and craniofacial parameters: A forensic view published in the Journal of Forensic Legal Medicine by authors S.K. Buyuk *et al.* in the year 2017, ^6^ the correlation coefficient derived/reported was 0.231. With an alpha error of 5% and a power of 80%, the Z values of the given alpha and beta values are 1.95996398454005 and 0.841621233572915. With the correlation coefficient and using the above formula the required sample size was 142.

### Participants

The frontal sinuses were evaluated in patients who were above 20 years with no history of trauma, cranial anomalies, associated pathologies in the sinus, skull/orthognathic surgery, and endocrine disorders. 66 scans were of the male sex and 77 scans were of the female sex in the age range of 20-54 years old.

### Data collection

The cone beam computed tomographic (CBCT) scans were acquired using the Promax 3DMid (Planmeca Oy., Helsinki, Finland) CBCT unit. The large FOV, low dose images that allowed complete visualization above the frontal sinuses were taken with the exposure parameters being 90 kVp, 5.6 mA, and exposure time of 18 seconds (DAP-925 mGy*cm2, CTDI-3.7 mGy). A slice thickness of 0.400 mm was used to assess the sections. The exposure parameters according to the standard default values were based on the FOV. The images were reconstructed using the Romexis software version 4.6.2.R ( ITK-SNAP 4.0 I can be used as an alternative).

### Measurements

**Figure 1.**
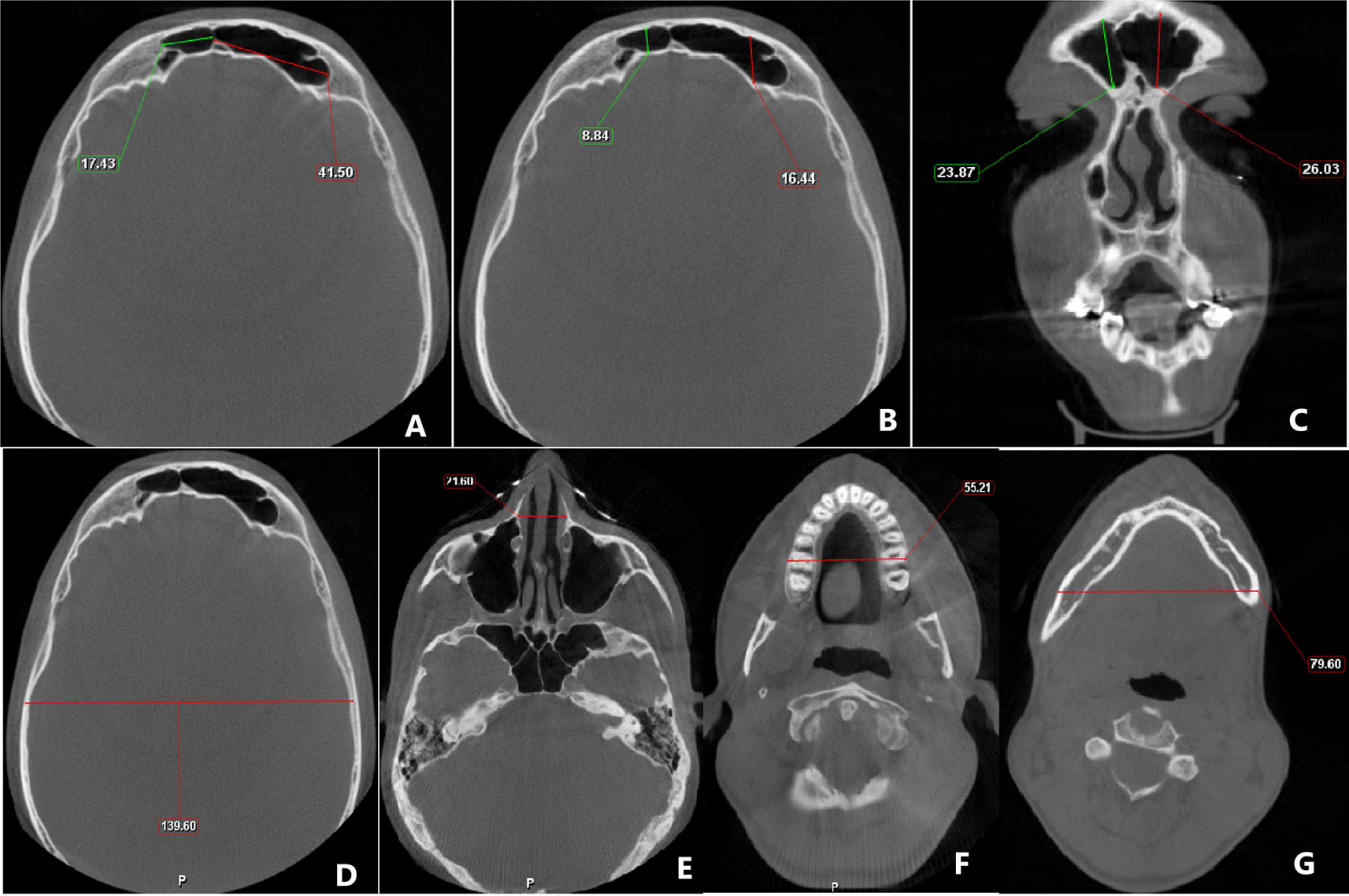
Cone-beam computed tomography image showing the measurements of A) mesio-distal dimension of frontal sinus; B) antero-posterior measurement of frontal sinus; C) supero-inferior measurement of frontal sinus; D) cranial width; E) nasal width; F) maxillary width; G) mandibular width.

The linear measurements were done on the reconstructed image in the following way ( Figure 1):

• **Frontal sinus** - For assessment of maximum supero-inferior measurements, the coronal section was utilized and measurement was taken between the highest and lowest point of both the right and left frontal sinus, while the axial section was used to measure the maximum antero-posterior and mesiodistal width of the left and right frontal sinus.
• **Cranium** - For the measurement of the maximum width of the cranium, the axial section was adjusted to the level of the superior border of the orbit (while looking at the coronal view), and at this level, measurement was taken from the inner cortical plate on one side to the inner cortical plate on the contralateral side.
• **Nasal** - The maximal nasal width was taken in the axial section after adjusting it at the level of the zygomatic arch.
• **Maxilla** - Axial section was skimmed till the maximal width of the maxilla was observed and in this section, measurement was taken.
• **Mandibular width at antegonial notch** - The maximum width of the mandible was taken at the level of the antegonial notch. For this purpose, first, the axial view was angulated so that in the sagittal section, the gonial and antegonial notch were observed. The axial view was then adjusted to the level of the antegonial notch and in this section, the maximal mandibular width was taken.

### Statistical analysis

Data were analyzed using Statistical Product and Service Solutions (SPSS, version 20.0 (SPSS Inc, Chicago IL). Asymmetry between the dimensions measured was compared using paired t-test. The correlation of the anthropometric variables with the sinus morphometry was done using Spearman's correlation. The difference in the dimensions between the sexes was explored using an independent t-test and the precision of classification was calculated using discriminant function analysis.

## Results

There was asymmetry detected of the frontal sinus in all the patients in terms of the mesiodistal, anteroposterior and superoinferior dimensions with the majority showing the left side larger than the right side. ^24^ However, only the anteroposterior dimension was significantly more on the left side in males (p-value 0.012, paired-test) ( Table 1).

**Table 1.** Paired t-test to compare the frontal sinus on either side in both sexes.

		N	Mean ± SD	Mean difference ± SD	t	P value
Male						
Pair 1	Mesiodistal L	65	30.96±7.7	0.58±9.86	0.48	0.635
	Mesiodistal R	65	30.37±7.6			
Pair 2	Anterioposterior L	65	12.01±4.64	1.26±3.92	2.59	**0.012**
	Anterioposterior R	65	10.75±2.68			
Pair 3	Superioinferior L	65	29.49±6.14	0.9±4.78	1.51	0.135
	Superioinferior R	65	28.59±5.14			
Female						
Pair 1	Mesiodistal L	77	29.36±9	0.47±10.91	0.38	0.707
	Mesiodistal R	77	28.89±8.57			
Pair 2	Anterioposterior L	77	9.16±2.82	-0.22±2.8	-0.68	0.499
	Anterioposterior R	77	9.37±3.5			
Pair 3	Superioinferior L	77	26.99±5.83	0.61±4.66	1.16	0.252
	Superioinferior R	77	26.37±6.03			

L: left, R: right, N: number, SD: standard deviation.

Red and underline signifies that P value <0.05 are statistically significant.

Mandibular parameter correlates moderately with anteroposterior dimension on the left and right side in males but not in females. The anteroposterior dimension of the left side and the mesiodistal dimension of the left side correlate significantly (r=0.357, p=0.003) in males but not in females. On the other hand, the anteroposterior dimension of the right side correlates with the mesiodistal dimension of the right side in both females and males. The cranial parameter shows a moderate negative correlation with the anteroposterior dimension of the left side (r value of-0.235, p-value of 0.039) and nasal dimension (r value of -0.248, p-value of 0.03) in females but not in males. Maxillary dimension showed a significant positive correlation with mandible (r value of 0.326 and p value of 0.004) in females and not in males. Nasal dimensions correlated with superoinferior dimensions on the right side in females (0.233 and p-value of 0.041) but not in males. Superoinferior dimensions correlated positively with mesiodistal as well as anteroposterior dimensions in males and females (p<0.05, r ranges from 0.248 to 0.461) ( Table 2).

**Table 2.** Spearman’s correlation between either sexes.

	Mesio-Distal L	Mesio-Distal R	Anterio-Posterior L	Anterio-Posterior R	Superio-Inferior L	Superio-Inferior R	Nasal	Mandible	Cranial
Male									
Mesio-distal R	0.192 (0.125)								
Anterio-posterior L	.357 ** (0.003)	0.223 (0.074)							
Anterio-posterior R	0.134 (0.287)	.282 * (0.023)	.644 ** (<0.001)						
Superio-inferior L	.381 ** (0.002)	0.231 (0.064)	.312 * (0.012)	0.178 (0.155)					
Superio-inferior R	.431 ** (<0.001)	.285 * (0.021)	.336 ** (0.006)	.248 * (0.046)	.629 ** (<0.001)				
Nasal	0.08 (0.527)	0.214 (0.087)	-0.076 (0.548)	0.209 (0.094)	0.047 (0.707)	0.218 (0.081)			
Mandible	0.206 (0.099)	0.102 (0.419)	.262 * (0.035)	.271 * (0.029)	-0.066 (0.603)	0.069 (0.585)	0.167 (0.183)		
Cranial	0.032 (0.803)	-0.159 (0.206)	0.109 (0.389)	0.119 (0.344)	-0.029 (0.818)	0.013 (0.916)	-0.147 (0.243)	-0.125 (0.322)	
Maxilla	0.026 (0.839)	0.14 (0.265)	-0.051 (0.684)	0.02 (0.877)	0.117 (0.352)	0.087 (0.489)	.419 ** (0.001)	0.193 (0.123)	-0.141 (0.262)
Female									
Mesio-distal R	.248 * (0.03)								
Anterio-posterior L	0.1 (0.388)	0.164 (0.153)							
Anterio-posterior R	0.122 (0.291)	.341 ** (0.002)	.454 ** (<0.001)						
Superio-inferior L	.348 ** (0.002)	.439 ** (<0.001)	.249 * (0.029)	.334 ** (0.003)					
Superio-inferior R	0.221 (0.054)	.468 ** (<0.001)	.313 ** (0.006)	.461 ** (<0.001)	.684 ** (<0.001)				
Nasal	-0.002 (0.988)	0.101 (0.383)	0.098 (0.399)	0.203 (0.076)	0.159 (0.168)	.233 * (0.041)			
Mandible	0.049 (0.671)	-0.021 (0.858)	-0.014 (0.905)	0.073 (0.526)	-0.042 (0.716)	-0.02 (0.86)	-0.021 (0.857)		
Cranial	-0.16 (0.164)	-0.062 (0.591)	-.235 * (0.039)	-0.138 (0.232)	-0.143 (0.213)	0.021 (0.855)	-.248 * (0.03)	0.06 (0.602)	
Maxilla	-0.073 (0.529)	0.059 (0.612)	0.209 (0.069)	.286 * (0.012)	0.029 (0.801)	0.064 (0.579)	0.025 (0.831)	.326 ** (0.004)	0.038 (0.743)

* Significant with p value <0.05.

** Significant with p value <0.001.

An independent t-test was done to compare the measurements between the two sexes and it was found that there was a statistically significant difference between them. The frontal sinus, nasal, cranial, maxillary and mandibular widths were higher in males when compared to the females and this was statistically significant except for the nasal width, cranial and mesiodistal measurements of the frontal sinus ( Table 3).

**Table 3.** Independent t-test to compare values between males and females.

	Male (n=65)	Female (n=77)	t	P value
	Mean ± sd	Mean ± sd		
Mesiodistal L	30.96±7.7	29.36±9	1.124	0.263
Mesiodistal R	30.37±7.6	28.89±8.57	1.08	0.282
Anterioposterior L	12.01±4.64	9.16±2.82	4.324	**<0.001**
Anterio posterior R	10.75±2.68	9.37±3.5	2.587	**0.011**
Superioinferior L	29.49±6.14	26.99±5.83	2.485	**0.014**
Superioinferior R	28.59±5.14	26.37±6.03	2.334	**0.021**
Nasal	24.65±2.73	24.23±2.66	0.933	0.353
Mandible	80.98±6.86	76.2±5.21	4.604	**<0.001**
Cranial	129.98±16.05	127.26±6.59	1.362	0.175
Maxilla	60.4±4.41	58.27±4.11	2.976	**0.003**

Red and underline signifies that P value <0.05 are statistically significant.

Comparing the discriminant function scores we found that the male values are consistently higher than the female values in all the cases. The discriminant function equations and the demarcation points are given in Table 4. The highest accuracy was seen with the mandibular width with 68.3% accuracy, followed by anteroposterior left dimensions with 66.90%. Both the mandible and the left anterior-posterior dimensions were more accurate in females than in males. The other variables like the superio-inferior and mesio-distal measurements of frontal sinus and nasal dimensions were below 60% accuracy. The discriminant function equations can be used with sectioning points of roughly zero to demarcate the sex.

**Table 4.** Discriminant function analysis for sex.

Parameter	Male mean values	Male SD	Female mean values	Female SD	Equation	Percentage of females correctly classified	Percentage of males correctly classified	overall accuracy	Male centroid	Female centroid	Sectioning point	Demarcating point
Mesio-distal L	30.9562	7.69745	29.3599	9.00332	Discriminant function (D)=-3.569+(0.119) x (Mesiodistal L)	61.5	54.5	57.70%	0.103	-0.087	-2.8169E-05	29.9916
Mesio-distal R	30.3722	7.60354	28.8914	8.57115	Discriminant function (D)=-3.631+(0.123) x (Mesiodistal R)	49.2	62.3	56.30%	0.099	-0.083	0.000309859	29.52033
Anterio-posterior L	12.0088	4.64454	9.1569	2.81874	Discriminant function (D)=-2.779+(0.266) x (Anterioposterior L)	58.5	74	66.90%	0.411	-0.347	-2.8169E-05	10.44737
Anterio-posterior R	10.7475	2.6839	9.374	3.49745	Discriminant function (D)=-3.174+(0.317) x (Anterioposterior R)	55.4	70.1	63.40%	0.236	-0.199	0.000119718	10.01262
Superio-inferior L	29.4866	6.13768	26.9856	5.83334	Discriminant function (D)=-4.708+(0.167) x (Superioinferior L)	58.5	59.7	59.20%	0.227	-0.192	-0.000204225	28.19162
Superio-inferior R	28.5909	5.14431	26.3719	6.0325	Discriminant function (D)=-4.853+(0.177) x (Superioinferior R)	55.4	58.4	57.00%	0.213	-0.18	-0.000105634	27.41808
Nasal	24.6549	2.72772	24.2325	2.65697	Discriminant function (D)=-9.082+(0.372) x (Nasal)	52.3	53.2	52.80%	0.085	-0.072	-0.000133803	24.41398
Mandible	80.9786	6.86311	76.199	5.21295	Discriminant function (D)=-13.013+(0.166) x (Mandible)	67.7	68.8	68.30%	0.43	-0.363	-7.04225E-06	78.39157
Cranial	129.9837	16.04581	127.2568	6.58519	Discriminant function (D)=-10.813+(0.084) x (Cranial)	69.2	57.1	62.70%	0.124	-0.105	-0.000176056	128.7262
Maxilla	60.404	4.41019	58.2729	4.11417	Discriminant function (D)=-13.934+(0.235) x (Maxilla)	67.7	55.8	61.30%	0.272	-0.229	0.000330986	59.29362

## Discussion

Sex, age and stature are crucial factors in identifying unknown individuals through skeletal remains, especially when the body is decomposed or fragmented beyond recognition. Radiographs are often used to aid in this process, as the anatomic structures and their variations are unique to each individual. ^7^ In forensic dentistry, craniometric parameters also play a significant role in both postmortem and antemortem evaluations of bodies. Cranial dimensions vary among different populations, and measuring the cranium can help determine racial differences. ^8^ Zuckerkandl reported in 1895 that the morphology of the frontal sinus is unique to each individual and can be used for identification purposes. ^9^ Our study was a retrospective cone beam computed tomographic study, utilizing archived images from the South-Indian population, specifically a subset of the Dakshina Kannada district on the West coast of India. This research aims to establish specific anthropometric patterns for different populations, highlighting the importance of considering regional variations in forensic investigations.

The frontal sinus is a structure that becomes visible on radiographs at around 5-6 years of age and stops growing around 20 years. ^7^ It is intriguing to note that no two individuals have the same frontal sinus. ^10^ Research has shown that the frontal sinus is larger in males than in females, ^11^^–^^13^ which is consistent with our own findings. Moreover, when comparing the size of the frontal sinus on each side, we observed that the left side was larger than the right side. This finding is in line with previous studies conducted by Rubira-Bullen IR *et al.* (2010), Kanat A *et al.* (2015), Soman BA *et al.* (2016), and Denny C *et al.* (2018), ^12^^–^^16^ but not with the study by Camargo *et al.* 2007. ^11^ The difference in size could be attributed to the independent development of the sinuses, resulting in varying sizes even among individuals of the same age. ^14^ Kanat *et al.* (2015) ^15^ suggested that handedness and footedness may play a role in predicting cerebral dominance, with right-handed individuals exhibiting left hemispheric dominance and vice versa for left-handed individuals.

In summary, the frontal sinus is a distinctive anatomical structure that exhibits variations in size and shape among individuals. Our study corroborates previous research indicating that males tend to have larger frontal sinuses than females, and that the left side is typically larger than the right. These differences may be attributed to independent development and handedness/footedness, which could potentially impact cerebral dominance.

Our study found that the maximal cranial width measurement was comparable to the research conducted by Ulcay T and Kamaşak B (2021). ^17^ Additionally, we discovered that cranial width was significantly greater in males than in females. ^18^^,^^19^ This dimorphism in the male skull is attributed to pubertal changes that result in increased muscular attachments, while the female skull retains juvenile features. ^20^

According to a study conducted by Buyuk SK (2017), it was discovered that males have a greater maxillary width than females. ^6^ Dr. G Venkat Rao and G Kiran (2016), stated in their article that dental arch width is indicative of the size of the basal bone. As the basal bone of the jaws is larger in males, this explains why males have a larger maxillary arch. ^21^

The width of the mandible was found to be greater in males, which is consistent with the findings of previous studies conducted by Sreelekha D *et al.* (2020) ^22^ and Albalawi AS *et al.* (2019). ^3^

The present study investigated nasal width in both males and females and determined that there were no significant differences between the two groups. This finding is consistent with previous research conducted by Buyuk SK (2017) and M. I. Marini *et al.* (2020). ^6^^,^^23^

Spearman's correlation analysis reveals that there is a significant correlation between the right side anterolateral and mesiodistal/superior inferior dimensions in both males and females. Additionally, mandibular dimensions show a significant correlation with anteroposterior dimensions in males, while cranial dimensions exhibit a negative correlation with anteroposterior and nasal dimensions in females. Furthermore, maxillary dimensions display a significant correlation with anteroposterior and mandibular dimensions in females.

These observations can provide valuable insights for future research by comparing findings across different ethnicities and nationalities. However, to ensure a more accurate representation of the population, it is necessary to conduct further studies with larger sample sizes.

## Conclusions

Cone beam computed tomography has revolutionized the way we approach diagnostic imaging, providing a more comprehensive and accurate view of the head and neck region. Its benefits extend beyond the medical field, making it an essential tool for forensic investigations. With its increasing popularity, CBCT is sure to continue to play a vital role in the future of diagnostic imaging.

This study has the potential to offer valuable insights to medical professionals and researchers in the field of craniofacial medicine. By leveraging advanced imaging technology and focusing on a specific population, we can gain a deeper understanding of the intricate relationship between the frontal sinus and craniofacial parameters.

## Data Availability

figshare: Investigating the Link between Frontal Sinus Morphology and Craniofacial Characteristics with Sex: A 3D CBCT study on the South Indian Population. https://doi.org/10.6084/m9.figshare.23559849.v1. ^24^ This project contains the underlying measurement data (please note code 1 represents male, and 2 represents female in the sex column). Data are available under the terms of the Creative Commons Attribution 4.0 International license (CC-BY 4.0).
