# Supplementary material for: Investigating the link between frontal sinus morphology and craniofacial characteristics with sex: A 3D CBCT study on the South Indian population
Source: F1000Res. 2023 Sep 11;12:811. Originally published 2023 Jul 11. [Version 2] doi: 10.12688/f1000research.137008.2 (PMC10511843; doi:10.12688/f1000research.137008.2)
Supplement: Peer review history

# Reviewer report: Reviewer response for version 2

**DOI:** 10.5256/f1000research.154636.r205338

**Published:** 2023-09-20

**Authors:** Arvind Babu Rajendra Santosh

**Affiliations:** 1 Faculty of Medical Sciences, Mona Campus, The University of The West Indies, Kingston, Jamaica

I am writing to provide my assessment of the revisions made by the authors in response to the reviewer comments for the manuscript titled "Investigating the link between frontal sinus morphology and craniofacial characteristics with sex: A 3D CBCT study on the South Indian population."

 In continuation of a previous comment regarding the title that mentions "with sex," I recommend that the objective should explicitly state the investigation of gender-related variations in frontal sinus morphology. Therefore, I suggest that the purpose be revised as follows:

 "The purpose of this study is to evaluate the morphological structure of the frontal sinuses, compare them with the nasal, maxillary, cranial, and mandible widths, and investigate gender-related variations in these characteristics among the South Indian population."

 After carefully reviewing the revised version, I am pleased to report that the authors have satisfactorily addressed all the concerns raised during the peer review process. The aforementioned comment can be revisited, and if the authors agree with the suggestion, they can revise the purpose of the study accordingly. With that said, I support the indexing of the manuscript, either in its current form or with the revised purpose of the study.

Is the work clearly and accurately presented and does it cite the current literature?

Yes

If applicable, is the statistical analysis and its interpretation appropriate?

I cannot comment. A qualified statistician is required.

Are all the source data underlying the results available to ensure full reproducibility?

Yes

Is the study design appropriate and is the work technically sound?

Yes

Are the conclusions drawn adequately supported by the results?

Yes

Are sufficient details of methods and analysis provided to allow replication by others?

Yes

Reviewer Expertise:

Primary focus is on oral disease, and secondary attention is given towards forensic dentistry

I confirm that I have read this submission and believe that I have an appropriate level of expertise to confirm that it is of an acceptable scientific standard.

# Reviewer report: Reviewer response for version 1

**DOI:** 10.5256/f1000research.150156.r186253

**Published:** 2023-07-27

**Authors:** Arvind Babu Rajendra Santosh

**Affiliations:** 1 Faculty of Medical Sciences, Mona Campus, The University of The West Indies, Kingston, Jamaica

The researchers undertook an investigation into the essential areas of forensic dental sciences, particularly focusing on the morphometric characterization of the frontal sinus with respect to gender.

1. The title of the study clearly highlights this specific area of interest. However, in the last two paragraphs of the introduction, it would be advisable for the researchers to provide a more comprehensive justification for studying the morphometric characterization of the frontal sinus with consideration of gender.
2. The study's methodology was well-structured and comprehensive, facilitating reliable data collection. However, for the data analysis section, I recommend seeking input from a statistician. Did the study use age-gender matching in their sampling strategy?
3. The most highlighting and promising results of the study should be emphasized in the initial section of discussion.
4. Add a statement that directly reflects striking findings of your study in the conclusion that answer your hypothesis. 

Overall, this study is a valuable contribution to the literature, and we commend the authors for their excellent work. I endorse the manuscript for indexing with minor revisions.

Is the work clearly and accurately presented and does it cite the current literature?

Yes

If applicable, is the statistical analysis and its interpretation appropriate?

I cannot comment. A qualified statistician is required.

Are all the source data underlying the results available to ensure full reproducibility?

Yes

Is the study design appropriate and is the work technically sound?

Yes

Are the conclusions drawn adequately supported by the results?

Yes

Are sufficient details of methods and analysis provided to allow replication by others?

Yes

Reviewer Expertise:

Primary focus is on oral disease, and secondary attention is given towards forensic dentistry

I confirm that I have read this submission and believe that I have an appropriate level of expertise to confirm that it is of an acceptable scientific standard, however I have significant reservations, as outlined above.

# Reviewer report: Reviewer response for version 1

**DOI:** 10.5256/f1000research.150156.r186254

**Published:** 2023-07-19

**Authors:** Sudeendra Prabhu

**Affiliations:** 1 Department of Oral Pathology, Forensic Odontology, Yenepoya Dental College, Yenepoya (Deemed to be University), Mangalore, Karnataka, India

- Good study and well-written manuscript.
- But the discussion and conclusion is incomplete. Kindly mention about the importance of parameters taken for sexual dimporphism and its correlation with sex in the CBCT.
- Highlight the important parameter having good co-relation co-efficient.
- Kindly quote a few more studies using CBCT for sex dimorphism.
- This manuscript can be accepted for indexing with minor modifications.

Is the work clearly and accurately presented and does it cite the current literature?

Yes

If applicable, is the statistical analysis and its interpretation appropriate?

Yes

Are all the source data underlying the results available to ensure full reproducibility?

Yes

Is the study design appropriate and is the work technically sound?

Yes

Are the conclusions drawn adequately supported by the results?

No

Are sufficient details of methods and analysis provided to allow replication by others?

Yes

Reviewer Expertise:

oral pathology

I confirm that I have read this submission and believe that I have an appropriate level of expertise to confirm that it is of an acceptable scientific standard.
